# IL-33-ST2 signaling in fibro-adipogenic progenitors alleviates immobilization-induced muscle atrophy in mice

**DOI:** 10.1186/s13395-024-00338-2

**Published:** 2024-04-01

**Authors:** Yoshiyuki Takahashi, Masaki Yoda, Osahiko Tsuji, Keisuke Horiuchi, Kota Watanabe, Masaya Nakamura

**Affiliations:** 1 Department of Orthopaedic Surgery, Keio University School of Medicine, 35 Shinanomachi, Shinjuku-Ku, Tokyo, 160-8582 Japan; 2 Department of Orthopedic Surgery, National Defense Medical College, Namiki 3-2, Tokorozawa, Saitama 359-8513 Japan

**Keywords:** IL-33, ST2, Fibro-adipogenic progenitors, Immobilization, Cell sorting, Flow cytometry, Mouse, Muscle atrophy, Skeletal muscle

## Abstract

**Background:**

The regenerative and adaptive capacity of skeletal muscles reduces with age, leading to severe disability and frailty in the elderly. Therefore, development of effective therapeutic interventions for muscle wasting is important both medically and socioeconomically. In the present study, we aimed to elucidate the potential contribution of fibro-adipogenic progenitors (FAPs), which are mesenchymal stem cells in skeletal muscles, to immobilization-induced muscle atrophy.

**Methods:**

Young (2–3 months), adult (12–14 months), and aged (20–22 months) mice were used for analysis. Muscle atrophy was induced by immobilizing the hind limbs with a steel wire. FAPs were isolated from the hind limbs on days 0, 3, and 14 after immobilization for transcriptome analysis. The expression of ST2 and IL-33 in FAPs was evaluated by flow cytometry and immunostaining, respectively. To examine the role of IL-33-ST2 signaling in *vivo*, we intraperitoneally administered recombinant IL-33 or soluble ST2 (sST2) twice a week throughout the 2-week immobilization period. After 2-week immobilization, the tibialis anterior muscles were harvested and the cross-sectional area of muscle fibers was evaluated.

**Results:**

The number of FAPs increased with the progression of muscle atrophy after immobilization in all age-groups. Transcriptome analysis of FAPs collected before and after immobilization revealed that *Il33* and *Il1rl1* transcripts, which encode the IL-33 receptor ST2, were transiently induced in young mice and, to a lesser extent, in aged mice. The number of FAPs positive for ST2 increased after immobilization in young mice. The number of ST2-positive FAPs also increased after immobilization in aged mice, but the difference from the baseline was not statistically significant. Immunostaining for IL-33 in the muscle sections revealed a significant increase in the number of FAPs expressing IL-33 after immobilization. Administration of recombinant IL-33 suppressed immobilization-induced muscle atrophy in aged mice but not in young mice.

**Conclusions:**

Our data reveal a previously unknown protective role of IL-33-ST2 signaling against immobilization-induced muscle atrophy in FAPs and suggest that IL-33-ST2 signaling is a potential new therapeutic target for alleviating disuse muscle atrophy, particularly in older adults.

## Introduction

Muscle wasting or atrophy can result from a range of conditions, such as injury, immobility, chronic inflammatory disease, and cachexia [1–3]. In most cases, muscle atrophy is a fully reversible condition. However, the exceptional regenerative and adaptive capacity of skeletal muscles reduces with age, leading to severe disability and frailty in older adults [4, 5]. To date, there are no established treatment modalities or medications—other than physical exercise or nutritional support—to facilitate recovery from muscle atrophy [6, 7]. Considering that most developed countries are rapidly aging societies, developing treatments to ameliorate this undesirable condition is both medically and socioeconomically important.

Skeletal muscles have at least two distinct types of stem cells: muscle satellite cells (SCs), which can differentiate into muscle fiber cells, and fibro-adipogenic progenitors (FAPs), which can differentiate into multiple cell lineages, including osteoblasts, chondrocytes, and adipocytes. SCs are the sole stem cells that can differentiate into myoblasts and are indispensable for muscle regeneration after injury [8]; however, previous studies have shown that SCs are not necessarily required for the homeostasis or adaptive capability of muscle fibers, as evidenced in several different mouse models lacking SCs [9–11]. In contrast, FAPs are considered the source cells of various skeletal muscle conditions, such as fatty infiltration, heterotopic ossification, and fibrosis. Recent studies have revealed that mice lacking FAPs develop progressive muscle atrophy and show a decrease in the number of SCs [12–14]. These studies indicate that FAPs are major regulators of SCs and muscle fibers and are essential for preserving skeletal muscle homeostasis and adaptability. In the present study, we aimed to elucidate the potential role of FAPs in immobilization-induced muscle atrophy.

Our findings show that the *Il33* and *Il1rl1* transcript, which encode the IL-33 receptor ST2, are transiently induced in FAPs of young mice and, to a lesser extent, in aged mice following immobilization. Of note, we found that administration of recombinant IL-33 attenuates the progression immobilization-induced muscle atrophy in aged mice, but not in young mice, indicating that the IL-33-ST2 signaling is defective in aged mice and that IL-33 supplementation can have a positive effect. Taken together, the present study highlights the previously uncharacterized roles of FAPs and the IL-33-ST2 autocrine signaling in the regulation of immobilization-induced muscle atrophy.

## Methods

### Mouse models

C57BL/6 J male mice were purchased from CLEA Japan (Shizuoka, Japan) and Oriental Yeast (Tokyo, Japan). To induce muscle atrophy, bilateral hind limbs were immobilized for 2 weeks using a steel wire, as described previously [15]. The wire was replaced as needed during the 2-week immobilization period. In certain experiments, mice were injected intraperitoneally with recombinant mouse IL-33 (2 μg/100 μl PBS; BioLegend, San Diego, CA, USA) or soluble ST2 (sST2) (5 μg/100 μl PBS; 1004-MR-050; R&D Systems, Minneapolis, MN, USA) twice a week. The first injection to the hindlimb immobilization model was performed at the start of immobilization. B6N.Cg-Tg(Pdgfra-cre/ERT)467Dbe/J (018280) and B6.Cg-Gt(ROSA)26Sor^tm14(CAG−tdTomato)Hze^/J (007914) transgenic mice were purchased from Jackson Laboratories (Bar Harbor, ME, USA). These two transgenic mice were mated to generate platelet-derived growth factor receptor α (PDGFRα) reporter mice, in which PDGFRα-positive cells, including FAPs, express the tdTomato fluorescent protein upon tamoxifen induction. These mice were used to visualize FAPs in muscle sections (Fig. 3C). To induce Cre-mediated recombination, mice were intraperitoneally injected with tamoxifen (100 mg/kg; Toronto Research Chemicals, Toronto, Canada) dissolved in corn oil three times every other day.

### Flow cytometry

Skeletal muscles from the hind limbs were used for flow cytometric analysis. Single-cell isolation was performed as described previously [16]. In short, after removal of the fat tissues, vessels, nerves, and tendons, the muscles were minced with forceps and digested in Hanks’ balanced salt solution containing 0.2% collagenase type II (Worthington Biochemical, Lakewood, NJ, USA). The digested samples were filtered through 70- and 40-μm cell strainers to remove the debris. Red blood cells were removed using Red Blood Cell Lysis Buffer (Roche Diagnostics, Rotkreuz, Switzerland). The following fluorochrome-conjugated monoclonal antibodies were used for cell sorting: anti-CD31 (102,405, 1:50; BioLegend), anti-CD45 (103,107, 1:200; BioLegend), anti-PDGFRα (FAB1062P, 1:10; R&D Systems), anti-Sca1 (108,113, 1:80; BioLegend), anti-mouse ST2 (FAB10041A, 1:20; R&D Systems) and Brilliant Violet 421 streptavidin (405,226, 1:666; Biolegend). The biotinylated SM/C2.6 monoclonal antibody was generously provided by Dr. S. Fukada [17]. Flow cytometry was performed using the CytoFLEX S flow cytometer (Beckman Coulter, Brea, CA, USA). Because of the easier availability of young mice, more animals were used in the analysis for young mice than for adult and aged mice.

### Isolation and culture of SCs and FAPs

SCs (CD31^−^/CD45^−^/SM/C2.5^+^) and FAPs (CD31^−^/CD45^−^/Sca1^+^/PDGFRα^+^) were isolated using a MoFlo XDP cell sorter (Beckman Coulter). The isolated FAPs and SCs were cultured in high-glucose Dulbecco’s modified Eagle medium (DMEM) (Nacalai Tesque, Kyoto, Japan), 20% fetal bovine serum, and antibiotics in Matrigel (Corning, Corning, NY, USA)-coated dishes, as described previously [16]. To induce myogenic differentiation of SCs, the medium was replaced with DMEM supplemented with 2% horse serum and antibiotics.

### Total RNA sequencing

FAPs isolated from mice 3 days and 2 weeks after hind-limb immobilization were used to evaluate transcriptional changes in FAPs after immobilization in young and aged mice. FAPs collected from untreated mice were used as baseline controls. Two mice were used at each time point (6 young and 6 aged mice). The total RNA was extracted from cells using the RNeasy Micro Kit (Qiagen, Hilden, Germany) according to the manufacturer’s instructions. cDNA was prepared using a SMART-Seq v4 Ultra Low Input RNA kit and sequenced using a NovaSeq 6000 sequencer (Illumina, San Diego, CA, USA). After filtering genes with low expression levels or levels with high variation from each RNA sequence dataset, we screened genes with > twofold change compared with baseline controls. To determine the 10 DEGs with the largest fold change, genes with a maximum transcript per million of less than 10 transcripts in both the immobilized muscle samples and the corresponding baseline samples were excluded. The sequence data are available in the DDBJ Sequence Read Archive (https://www.ddbj.nig.ac.jp/) under accession number DRA016863.

### Quantitative RT-PCR

The tibialis anterior (TA) muscles from untreated and immobilized mice were harvested and minced. Total RNA was isolated using Sepasol-RNA I Super G reagent (Nacalai Tesque), according to the manufacturer’s instructions. Total RNA was reverse-transcribed using ReverTra Ace Reverse Transcriptase (Toyobo, Osaka, Japan). PCR and quantification were performed using the Thunderbird qPCR Mix (Toyobo) and a 7500 Real-Time PCR System (Applied Biosystems, Foster City, CA, USA). Gene transcript levels were normalized to the expression levels of *Actb* transcripts. The following primers were used: *Fbxo32* (forward: TCTCCAGACTCTCTACACATCC; reverse: GAATGGTCTCCATCCGATACAC), *Trim63* (forward: TACGTTGGTGCGAAATGAAA; reverse: AATCGCCAGTCACACAATGA), *Il-33* (forward: ACTATGAGTCTCCCTGTCCTG; reverse: ACGTCACCCCTTTGAAGC), *Il1rl1* (forward: TCTGTGGAGTACTTTGTTCACC; reverse: TCTGCTATTCTGGATACTGCTTTC), and *Actb* (forward: CTGAACCCTAAGGCCAACCGTG; reverse: GGCATACAGGGACAGCACAGCC). Because of the easier availability of young mice, more animals were used in the analysis for young mice than for aged mice.

### Cell staining

Cells were treated with mouse recombinant IL-33 (30 ng/ml) for 1 h, fixed with 4% paraformaldehyde, and permeabilized with 0.2% Triton X-100/PBS. Fixed cells were incubated with rabbit anti-NF-κB p65 (8242, 1:200; Cell Signaling Technology, Danvers, MA, USA) at 4 °C overnight, followed by secondary antibody staining with 4′,6-diamidino-2-phenylindole (DAPI). Images were captured using an Olympus FSX100 fluorescence microscope and Olympus FSX-BSW software (Olympus, Tokyo, Japan).

### Immunohistochemistry

To evaluate the cross-sectional area (CSA) of the muscle fiber, muscle tissues were snap-frozen in isopentane cooled with liquid nitrogen. Cryosections with thickness of 10 μm were fixed in acetone at − 20 °C for 20 min. Sections were washed with PBS and incubated with Blocking One (Nacalai Tesque) for 1 h, followed by incubation with rat anti-laminin-α2 (L0663, 1:500; Sigma-Aldrich, St. Louis, MO) at 4 °C overnight. Bound antibodies were detected using Alexa Fluor 488-conjugated anti-rat IgG (A21208, 1:1000; Invitrogen, Waltham, MA, USA). Images of entire sections were captured using an Olympus FSX100 fluorescence microscope and Olympus FSX-BSW software. The CSA of the muscle fibers was measured using ImageJ (National Institutes of Health, Bethesda, MD, USA).

Muscle tissues obtained from PDGFRα reporter mice were used for immunostaining of IL-33. Muscles were fixed in 2% paraformaldehyde for 2 h and consecutively immersed in 10% and 20% sucrose solutions for 1 h and 30% sucrose solution overnight. Muscles were snap-frozen in isopentane that was cooled with liquid nitrogen. Cryosections with thickness of 10 μm were rehydrated with PBS, permeabilized with 0.2% Triton X-100 for 10 min, and treated with proteinase K (1 μg/ml; Nacalai Tesque) for 5 min at room temperature. Sections were incubated with Blocking One for 1 h and subsequently with primary antibodies at 4 °C overnight. Goat anti-IL-33 antibody (AF3626-SP, 1:100; R&D Systems) and rabbit anti-RFP antibody (ab62341, 1:250; Abcam, Cambridge, UK) were used for immunostaining. For immunostaining of IL-33 in aged mice, sections of TA from wild-type mice were used and rabbit anti-PDGFRα antibody (sc338, 1:50; Santa Cruz Biotechnology, TX, USA) were used as primary antibody. Images were captured using an LSM980 confocal microscope (Carl Zeiss, Oberkochen, Germany). Three arbitrary areas (500 μm^2^) were chosen per sample, and cell counts were determined using Image J. For Hematoxylin and Eosin (H&E) staining, sections were fixed in acetone at − 20 °C for 20 min, washed in PBS and then stained in hematoxylin for 10 min and eosin for 7 min. The muscle sections were dehydrated in gradually increasing concentration of ethanol/ water solutions and fixed in 100% xylene.

### Statistical analysis

GraphPad Prism version 6.05 (GraphPad, La Jolla, CA, USA) was used for the statistical analyses. Student’s *t*-test (two-tailed and assuming equal variances) was used to calculate the *P* values. For comparison of more than two groups, one-way ANOVA was used, followed by Tukey’s post hoc testing. *P* < 0.05 was considered statistically significant. Data are presented as mean ± standard error of the mean unless otherwise noted.

## Results

### Immobilization leads to an increase in the number of FAPs

**Fig. 1:**
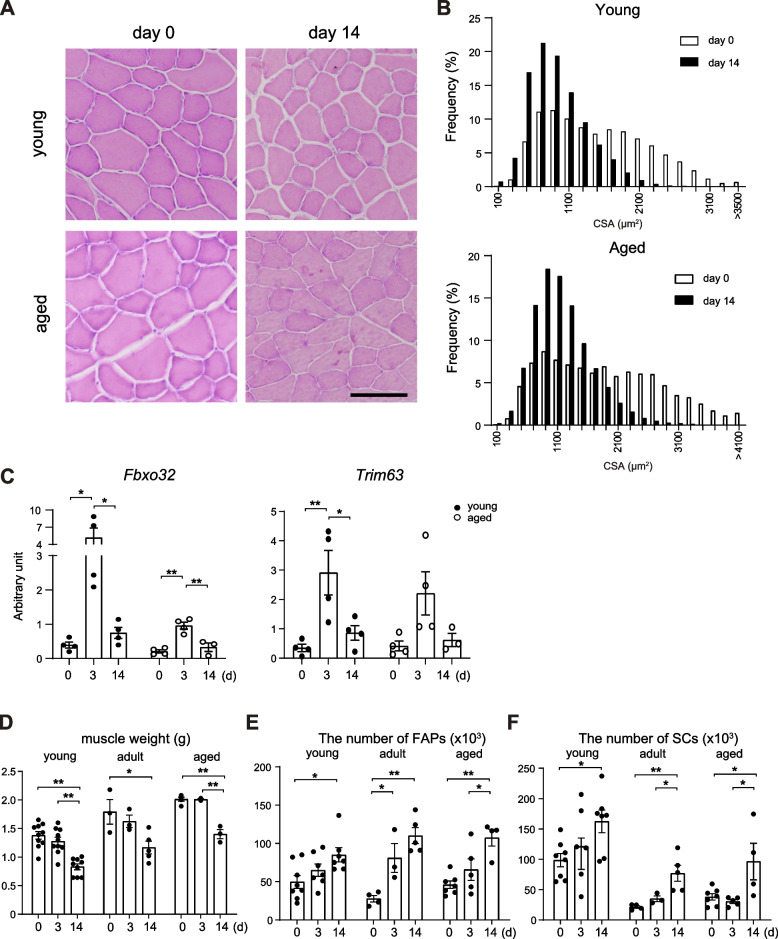
The number of FAPs and SCs in the TA muscle increases after hindlimb immobilization. **A** Micrographs of TA muscle tissue stained with H&E. Muscles were collected from days 0 and 14 after hindlimb immobilization of young (upper) and aged (lower) mouse. Bar, 50 μm. **B** Frequency distribution of CSA of muscle fibers of TA muscles collected from days 0 and 14 after hindlimb immobilization of young (upper) and aged (lower) mice. Accumulated data from three mice are shown in each group. **C** Expression levels of *Fbxo32* (left) and *Trim63* (right) transcripts in the TA muscles from young and aged mice on days 0, 3, and 14 of immobilization (*n* = 3–4). **D** Hindlimb muscle weight on days 3 and 14 after hindlimb immobilization in young, adult, and aged mice. **E**,**F** Number of FAPs (**E**) and SCs (**F**) on days 3 and 14 after hindlimb immobilization (*n* = 3–10 mice per group). *P < 0.05; **P < 0.01

To investigate the potential contribution of FAPs to muscle atrophy, we first determined whether the number of FAPs in skeletal muscles was affected by immobilization in mice. Immobilization-induced muscle atrophy was achieved by using a steel wire to immobilize the hind limbs, as described previously [15]. The hind limbs muscles were harvested for analysis on days 0, 3, and 14 after the treatment. The CSA of muscle fibers was reduced on day14 compared to day 0 (Fig. 1A, and B). Gene expression analysis using quantitative PCR confirmed that the levels of *Fbxo32* transcripts increased in the skeletal muscle after immobilization in both young and aged mice (Fig. 1C). As shown in Fig. 1D, the weight of the hind limbs muscle significantly decreased by day 14 in each age group. Next, we examined the number of FAPs and SCs in the hind limbsmuscle by flow cytometry after immobilization. Consistent with previous studies [14, 18], the numbers of SCs were lower in adult (12–14 months) and aged (20–22 months) mice than in young (2–3 months) mice at baseline (day 0; Fig. 1F, *P* < 0.01). Notably, our analysis revealed that the numbers of FAPs and SCs increased with the progression of muscle atrophy after immobilization in all groups. These observations suggest that FAPs and SCs play active roles in immobilization-induced muscle atrophy.

### Expression of ST2 and IL-33 transcripts increases in FAPs after immobilization

**Fig. 2:**
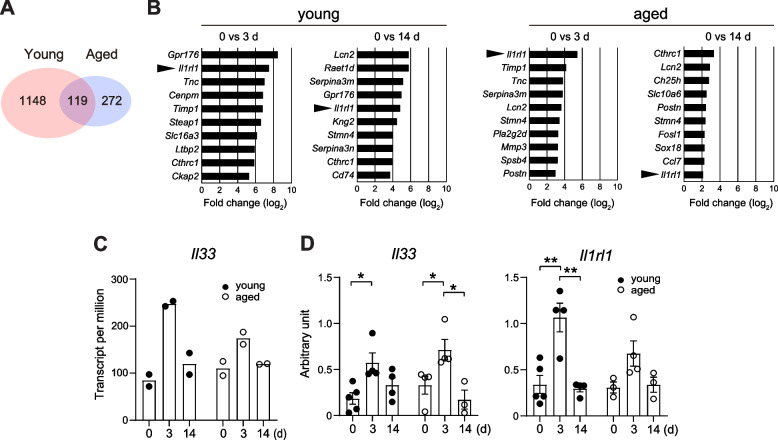
Expression of *Il33* and *Il1rl1* transcripts in FAPs is induced after immobilization. **A** Number of genes expressed in FAPs that showed > twofold or < 0.5-fold changes from the baseline controls on days 3 and 14 of hindlimb immobilization in young and aged mice. **B** Top 10 DEGs that showed the greatest increase from the baseline controls on days 3 and 14 of hindlimb immobilization in young and aged mice. **C** Transcript per million values of *Il33* in FAPs of young and aged mice on days 0, 3, and 14 of immobilization (*n* = 2). **D** Expression levels of *Il33* (left) and *Il1rl1* (right) transcripts in the TA muscles from young and aged mice on days 0, 3, and 14 of immobilization (*n* = 3–5). *P < 0.05; **P < 0.01

**Fig. 3:**
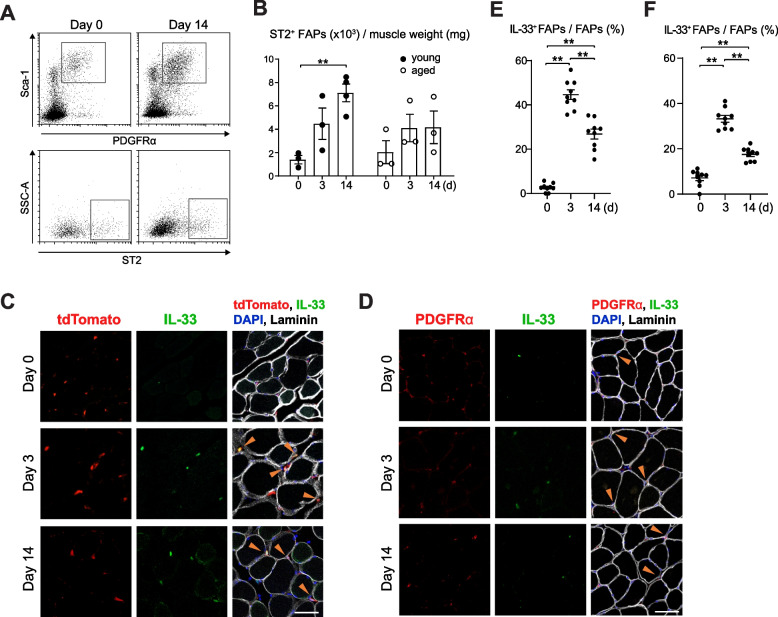
ST2-positive FAPs and IL-33-positive FAPs are induced upon immobilization. **A** Flow cytometric analysis of the FAPs population (upper) and ST2-positive FAPs (lower) using a single-cell suspension prepared from the muscles of young mice collected on days 0 and 14 of immobilization. Boxed areas indicate FAPs (upper) and ST2-positive FAPs (lower). **B** Number of ST2-positive FAPs per muscle weight in the TA muscles from young and aged mice collected on days 0, 3, and 14 of immobilization (*n* = 3). **C** Sections of the TA muscle of young mice stained for tdTomato (PDGFRα), IL-33, and laminin and with DAPI. Specimens were collected on days 0, 3 and 14 of immobilization. D Sections of the TA muscle of aged mice stained for PDGFRα, IL-33, and laminin and with DAPI. Specimens were collected on days 0, and 3 and 14 of immobilization. **E**, **F** Ratio of FAPs positive for IL-33 on days 0, 3 and 14 of immobilization in young (E) and aged (F) mice. Three random sections from each specimen (three mice) were evaluated. *P < 0.05; **P < 0.01. Bar, 50 μm

Considering that FAPs play an essential role in skeletal muscle homeostasis [12, 14], we evaluated the impact of immobilization on the gene expression of FAPs in young and aged mice. To accomplish this, we isolated FAPs from the hind limbs of young and aged mice on days 0, 3, and 14 post-immobilization and performed transcriptome analyses. Differentially expressed genes (DEGs) that exhibited more than a twofold change on days 3 and 14 compared with basal controls (day 0) were extracted; this analysis resulted in 1148 and 272 unique DEGs in young and aged mice, respectively. In total, 119 DEGs overlapped between the two groups (Fig. 2A). Figure 2B shows the 10 DEGs with the largest differences in their expression levels between days 0 and 3 or day 14 in each group of young and aged mice. Among these genes, *Il1rl1* shown by arrowheads, which encodes the IL-33 receptor ST2, was upregulated in both young and aged mice after immobilization, indicating its potential role in disuse muscle atrophy. Additionally, *Il33* was identified as a DEGs with a significant increase, particularly in young mice, after immobilization (Fig. 2C). Gene expression analysis using quantitative PCR confirmed that the levels of *Il33* and *Il1rl1* transcripts transiently increased in the skeletal muscle after immobilization in young mice (Fig. 2D). Consistent with the results of transcriptome analysis, flow cytometry revealed an increase in the number of FAPs positive for ST2 after immobilization in young mice (Fig. 3A and B). The number of ST2-positive FAPs also increased after immobilization in aged mice, but the difference from the baseline was not statistically significant. Immunostaining for IL-33 in the TA muscle sections obtained from young PDGFRα reporter mice on days 0, 3, and 14 revealed a significant increase in the number of FAPs expressing IL-33 (Fig. 3C and E). Immunostaining using aged wild-type mice similarly showed increased expression of IL-33 after immobilization, though the proportion of FAPs expressed IL-33 on days 3 and days14 was smaller than young mice (Fig. 3D and F, *P* < 0.01). These findings confirm that the expression of IL-33 and ST2 is induced in FAPs during muscle atrophy, and that this induction is suppressed in old mice compared to young mice.

### Administration of IL-33 suppresses immobilization-indued muscle atrophy in aged mice

**Fig. 4:**
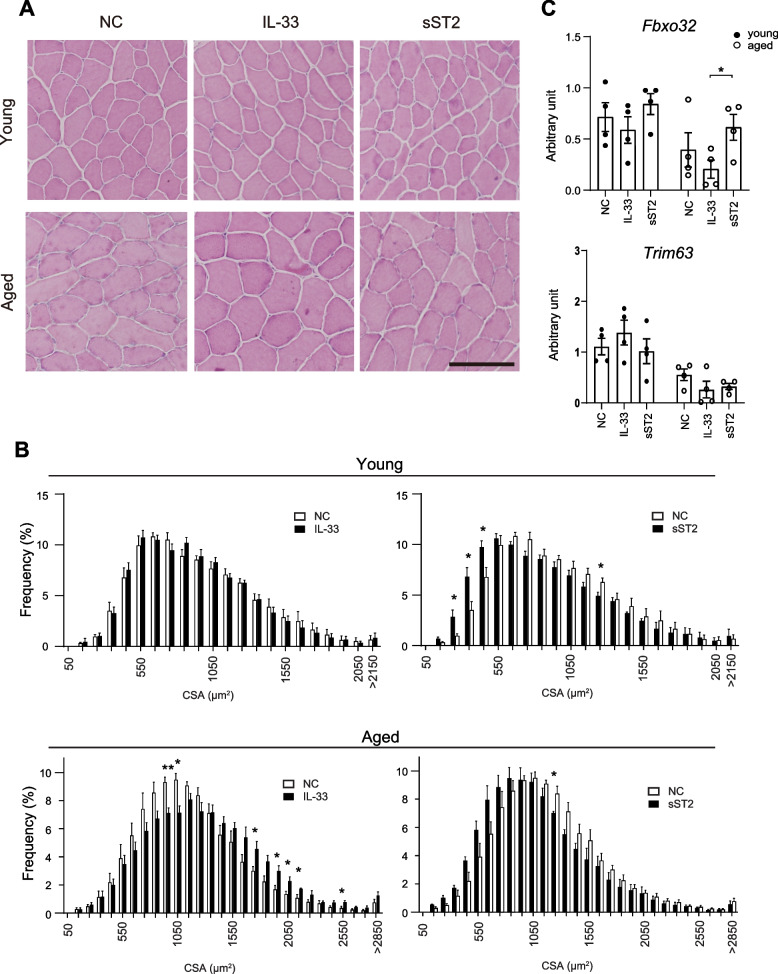
IL-33-ST2 signaling suppresses immobilization-induced muscle atrophy. **A** H&E staining of TA muscle cryosections at the day 14 after immobilization of negative-control, IL-33-treated and sST2-treated young (upper) and aged (lower) mice. Bar, 100 μm. B Frequency distribution of CSA of muscle fibers of TA muscles collected from IL-33- and sST2-treated young (upper) and aged (lower) mice. Accumulated data from four mice are shown in each group. **C** Expression levels of *Fbxo32* (upper) and *Trim63* (lower) transcripts in the TA muscles from young and aged mice on days 14 of immobilization (*n* = 4). NC, vehicle-treated negative control. *P < 0.05; **P < 0.01

The enhanced expression of IL-33 and its receptor ST2 in FAPs after immobilization, particularly in young mice, suggests that the IL-33-ST2 signaling pathway may have a protective effect against muscle atrophy. To test this hypothesis, we intraperitoneally administered recombinant IL-33 or sST2, which functions as a decoy receptor for IL-33, twice a week throughout the 2-week immobilization period and examined the impact of these recombinant proteins on muscle atrophy. After a 2-week period of immobilization, the TA muscles were harvested and analyzed for the CSA of the muscle fibers. IL-33 treatment had only a marginal effect on the progression of muscle atrophy in young mice (Fig. 4A, B). In contrast, it substantially suppressed muscle atrophy in aged mice, as demonstrated by the rightward shift of the CSA histogram in IL-33-treated mice (compared with vehicle-treated controls). Treatment with sST2 exacerbated muscle atrophy in both young and aged mice, as shown by the leftward shift of the CSA histogram in sST2-treated mice (compared with vehicle-treated controls). Gene expression analysis using quantitative PCR showed no significant difference among the levels of *Fbxo32* and *Trim63* transcripts in the skeletal muscle after IL-33 and sST2 administration in young mice. In contrast, the levels of *Fbxo32* transcript increased in sST2-treated mice compared with IL-33-treated mice in aged mice (Fig. 4C).

**Fig. 5:**
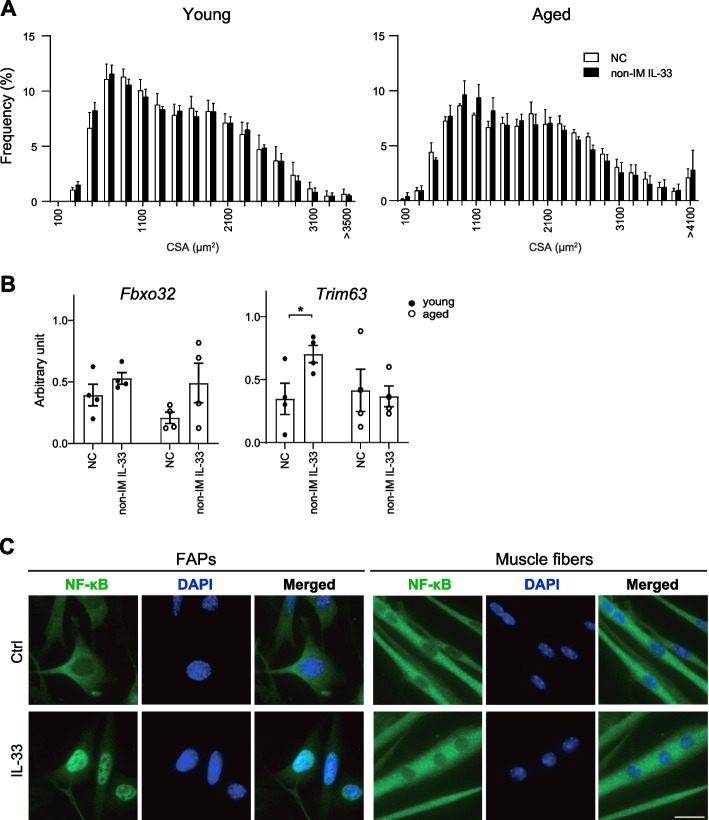
IL-33 does not affect muscle fibers in the non-immobilized state, but acts on FAPs. **A** Frequency distribution of CSA of muscle fibers of TA muscles collected from normal and IL-33-treated young (upper) and aged (lower) mice without hindlimb immobilization. Accumulated data from four mice are shown in each group. NC, vehicle-treated negative control; non-IM IL-33, non-immobilization IL-33-treated. **B** Expression levels of *Fbxo32* (left) and *Trim63* (right) transcripts in TA muscles from young and aged mice on day 14 of IL-33 administration without immobilization (*n* = 4). **C** FAPs and muscle fibers stained for NF-κB and with DAPI. Cells were treated with vehicle (Ctrl) or IL-33 for 1 h. Bar, 20 μm

To examine the effect of IL-33 on mice in normal condition, mice without hindlimb immobilization were treated with IL-33 intraperitoneally for 2 weeks. The TA muscles were harvested for CSA measurement after 2 weeks. There was no difference in the CSA of muscle fibers in the non-immobilized IL-33-treated group compared with those of the negative control group (Fig. 5A). These results indicate that IL-33 has no hypertrophic or atrophic effects on the muscle in its normal state. Gene expression analysis using quantitative PCR demonstrated no significant difference among the levels of *Fbxo32* transcripts in the skeletal muscle after administration of IL-33. In contrast, the levels of *Trim63 transcript* increased in non-immobilized IL-33-treated young mice compared with those in the negative control mice (Fig. 5B).Given that IL-33 counteracts muscle atrophy, we investigated whether IL-33 operates directly on muscle fibers or functions as an autocrine factor in FAPs. The FAPs and muscle fiber cells (prepared from SCs in vitro) were incubated with recombinant IL-33 for 1 h and stained for NF-κB, one of the intracellular molecules located downstream of ST2 [19]. We found that intranuclear translocation of NF-κB, which reflects the activation of ST2, was readily observed in FAPs but not in muscle fiber cells after IL-33 stimulation (Fig. 5C). Taken together, these results indicate that IL-33 functions primarily as an autocrine factor in FAPs and that IL-33-ST2 signaling plays a protective role against immobilization-induced muscle atrophy.

## Discussion

This study investigated the role of FAPs in muscle atrophy and identifies viable target molecules for the treatment of immobilization-induced muscle atrophy. We found that the number of FAPs increased after immobilization and that the expression of *Il1rl1* and *Il33* transcripts was highly induced in the FAPs of both young and aged mice after immobilization. Most importantly, we found that autocrine IL-33-ST2 signaling in FAPs plays a protective role against immobilization-induced muscle atrophy and that administration of recombinant IL-33 alleviated muscle atrophy in aged mice. These observations revealed a previously unknown contribution of FAPs in counteracting muscle atrophy and the unexpected involvement of IL-33-ST2 signaling in this process.

Although FAPs were initially identified as the cells responsible for muscle fat degeneration, heterotopic ossification, and fibrosis [20–22], subsequent studies have revealed that FAPs are crucial for skeletal muscle homeostasis, as evidenced by the severe muscle-wasting phenotype observed in FAP-depleted mice [12, 14]. However, their potential promoting and suppressive functions in muscle atrophy are poorly understood. Madaro et al*.* showed that STAT3-IL-6 signaling in FAPs is activated upon denervation and that inactivation of this signaling could restrain muscle atrophy in their mouse models, suggesting that FAPs promote denervation-induced muscle atrophy [23]. Similarly, Parker et al*.* found that *IL-1* transcripts were upregulated in FAPs in a hind-limb-immobilization model, suggesting its potential involvement in promoting muscle atrophy [24]. In agreement with these studies, our data indicate a significant increase in the number of FAPs as well as marked changes in the gene expression profile of FAPs after muscle atrophy. In contrast, although previous studies have suggested that FAPs promote muscle atrophy by secreting IL-6 and IL-1, our data indicate that FAPs mitigate immobilization-induced muscle atrophy via autocrine IL-33-ST2 signaling. The differences between our results and those of previous studies may be attributed to the variations in the mouse models used. Alternatively, the contribution of FAPs to muscle atrophy may differ depending on specific contexts and situations.

IL-33 is a member of the IL-1 cytokine family and plays multiple roles in innate and acquired immunity, such as the induction of Th2 cell maturation and activation of mast cells and natural killer cells [25]. With respect to its potential involvement in muscle biology, Kastenschmidt et al*.* reported that the IL-33 secreted by FAPs plays a role in the expansion of group 2 innate lymphoid cells and promotion of fibrosis in a mouse model of muscular dystrophy [26]. In contrast, Kuswanto et al*.* have shown that IL-33 is secreted by FAPs after muscle injury and regulates the muscle Treg cell population, thereby promoting muscle repair [27]. In addition, their study revealed that IL-33 production was decreased in aged mice, resulting in incomplete muscle repair after injury. The results obtained by Kuswanto et al*.* and our findings are in agreement with respect to the fact that IL-33 plays a protective role in maintaining muscle homeostasis and that IL-33-ST2 signaling is impaired in aged mice compared with young mice. Our study revealed that IL-33 alleviated muscle atrophy only in aged mice, whereas sST2 exacerbated muscle atrophy in both young and aged mice. These observations suggest that IL-33-ST2 signaling is saturated in young mice but is not adequately activated in aged mice after immobilization. If this is also the case in humans, IL-33 administration may be beneficial in suppressing disuse atrophy in the elderly; however, its efficacy in the young may be limited.

In this study, we observed intranuclear translocation of NF-κB in FAPs upon the administration of IL-33. We found that IL-33 acts protectively against muscle atrophy, although previous studies have shown that the activation of NF-κB is associated with muscle atrophy [28]. There are also reports showing that NF-κB contributes to hypertrophy of cardiac and skeletal muscle [29, 30], which suggest that the activation of NF-κB in FAPs does not necessarily cause muscle atrophy and that its functions are context dependent. Furthermore, it is possible that IL-33 does not cause muscle atrophy because it does not activate NF-κB in the muscle fibers themselves. This study had several limitations. First, changes in structural proteins and their encoding genes have not been investigated, and further analysis is needed. Second and most importantly, the molecular mechanism underlying the effect of IL-33 in suppressing muscle atrophy remains to be elucidated. As IL-33 does not appear to have a direct effect on muscle fiber cells, it is likely that FAPs suppress muscle atrophy by producing secretory factors that have a protective function against muscle atrophy. One potential contributing factor is WISP1, a secreted protein that enhances the regenerative potential of SCs [18]. Accordingly, our transcriptome analysis revealed that *Ccn4*, which encodes WISP1, was among the DEGs that were upregulated upon immobilization (data not shown). Our investigation of the genes induced by IL-33 in FAPs is ongoing, and we have identified several candidate genes that encode secretory factors with potential roles in maintaining muscle homeostasis. These findings will be investigated further and published in a separate study. In addition, considering that IL-33-ST2 signaling is a potential molecular target for suppressing disuse muscle atrophy in humans, determining whether this signaling pathway is activated in human FAPs after immobilization is important.

## Conclusions

Our data demonstrate that autocrine IL-33-ST2 signaling in FAPs protects against immobilization-induced muscle atrophy in mice. Most importantly, we found that IL-33 could alleviate disuse muscle atrophy in aged mice, suggesting a potential therapeutic application of this signaling pathway for the treatment of disuse muscle atrophy in the elderly, a condition for which no established medical intervention currently exists.

## Data Availability

All data generated or analyzed during this study are included in this published article. The datasets used and analyzed during the current study are available from the corresponding author on reasonable request. The sequence data are available in the DDBJ Sequence Read Archive (https://www.ddbj.nig.ac.jp/) under accession number DRA016863.

## Abbreviations

CSA: Cross-sectional area
DAPI: 4′,6-Diamidino-2-phenylindole
DEGs: Differentially expressed genes
DMEM: Dulbecco's modified Eagle medium
FAP: Fibro-adipogenic progenitor
HE: Hematoxylin and Eosin
SC: Satellite cell
PDGFRα: Platelet-derived growth factor receptor α
sST2: Soluble ST2
TA: Tibialis anterior
